# A Chemiluminescent Immunoassay for Osteocalcin in Human Serum and a Solution to the “Hook Effect”

**DOI:** 10.1155/2020/8891437

**Published:** 2020-12-09

**Authors:** Shuang Han, Yifeng Xue, Junlan Zhang, Jianrong Huang, Xiuxia Liu, Yankun Yang, Zhonghu Bai, Chunxin Wang

**Affiliations:** ^1^The Key Laboratory of Carbohydrate Chemistry and Biotechnology, Ministry of Education, School of Biotechnology, Jiangnan University, Wuxi 214122, China; ^2^National Engineering Laboratory for Cereal Fermentation Technology, Jiangnan University, Wuxi, China; ^3^Jiangsu Provincial Research Center for Bioactive Product Processing Technology, Jiangnan University, Wuxi 214122, China; ^4^The Affiliated Wuxi People's Hospital of Nanjing Medical University, Department of Laboratory Medicine, Wuxi, China

## Abstract

A chemiluminescent immunoassay for human serum osteocalcin, or bone Gla protein, was established using a double-antibody sandwich model. Examination of the hook effect revealed that it was significantly reduced, and no hook effect was observed at an osteocalcin concentration of 4000 ng/mL. The established method showed good analytical performance and thermal stability. The limit of detection was 0.03 ng/mL. The intra-assay coefficient of variation (CV) was 3.22%–5.64%, the interassay CV was 4.42%–7.25%, and the recovery rate was 93.22%–107.99%. Cross-reactivity (CR) was not observed with bovine, rat, mouse, rabbit, or porcine samples. The developed method showed a good correlation with the N-MID product from Roche. In total, 1069 clinical patient samples were measured using the reagent kit developed in this study.

## 1. Introduction

Osteocalcin is a vitamin K-dependent protein produced by osteoblasts in bones [1, 2]. It is the major noncollagenous protein of the bone matrix [3]; some osteocalcin is absorbed into the bone matrix after production, while the remainder is secreted into the blood [4]. Osteocalcin plays an important role in bone remodelling, specific expression, and regulation in osteoblasts [5, 6]. It is also known as a bone-related marker that is useful in diagnosing bone-related diseases, examining a bone's history, and monitoring the effects of interventions [7, 8]. Therefore, the development of an *in vitro* diagnostic assay for osteocalcin is particularly essential. The main forms of osteocalcin in serum include the entire molecule (amino acids 1–49) and the N-MID fragment (amino acids 43–49), the latter of which shows greater stability in the peripheral blood [9, 10]. The radiological immunoassay was the very first method for the analysis of osteocalcin, and the enzyme-linked immunosorbent assay was later established; however, both methods are time-consuming, labour-intensive, or harmful to the environment and human health. In this study, the hook effect was observed during the research process, as described by Lebeouf et al. [11], and several methods were employed to study and solve this problem. Finally, a double-antibody sandwich immunoassay was developed for the detection of serum osteocalcin in a chemiluminescent immunoassay (CLIA) system. Currently, CLIA is widely used on the market because it is a simple, sensitive, and cheap method for the high-throughput quantification of analytes in samples [12, 13].

## 2. Materials and Methods

### 2.1. Reagents and Materials

Dynabeads MyOne streptavidin-precoated beads, EZ-Link Sulfo-NHS-LC-Biotinylation Kit, succinimidyl 4-(N-maleimidomethyl)cyclohexane-1-carboxylate, and 4′-hydroxyazobenzene-2-carboxylic acid (HABA) solution were obtained from Thermo Fisher (Waltham, MA, USA); perfluorohexanoate, methanol, EDC, NHS, and osteocalcin were purchased from Sigma (St. Louis, MO, USA); horseradish peroxidase (HRP) was purchased from BBI Solutions (Portland, ME, USA); streptavidin was purchased from Hangzhou NeuroPeptide Biological Science and Technology Incorporation, Ltd. (China); the AKTA Purifier system was purchased from GE Healthcare (Chicago, IL, USA); microscopes were purchased from Olympus (Tokyo, Japan); auto-microplate chemiluminescent analyser was supplied by Baiming Biotechnology (Yancheng, China); and an auto-magnetic bead chemiluminescent analyser was supplied by Zecheng Biotechnology (Beijing, China). Antibodies were obtained from HyTest (Shanghai, China). Antigens were purchased from Sigma (St. Louis, MO, USA).

### 2.2. Reagent Component Preparation

#### 2.2.1. Antibody Conjugates with HRP

Different weights of anti-VD antibody and EDC were completely dissolved in separated 0.05 M sodium bicarbonate solutions; then, the solutions were mixed. HRP was dissolved in 0.1 M phosphate buffer (pH 7.2), the mixed solution was added, and the pH was lowered to 5.8 with diluted hydrochloric acid. Then, the solution was incubated for 5 h at 26°C. Finally, the impurities were removed with a desalination column.

#### 2.2.2. Characterisation of Antibody-HRP Conjugates

**Figure 1:**
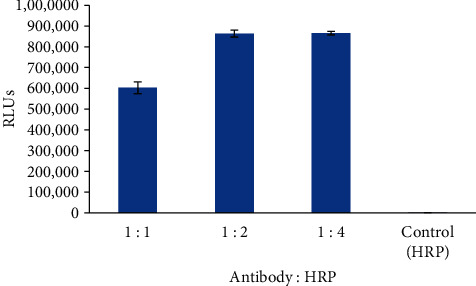
Characterisation of antibody-HRP conjugate (*n* = 3).

Osteocalcin was coated on microplates to verify the appropriate conjugation of the antibody and HRP. The antibody-HRP conjugate was prepared in different antibody/HRP ratios. Then, 50 *μ*L of antibody-HRP or HRP alone as a control was added to the precoated microplates, incubated for 30 min at 37°C, and washed 5 times with washing buffer. Next, 100 *μ*L of substrate reagent was added. The results indicated that the antibody-HRP conjugates were ready for use (Figure 1). As the histogram shows, relative light units (RLUs) increased along with the excess molar increase in HRP; when the HRP concentration was more than twofold that of the antibody, the signal showed little increase. Therefore, the antibody/HRP ratio was set at 1 : 2.

#### 2.2.3. Antibody Conjugates with Biotin

Antibody was dialysed into PBS buffer, pH 7.4. After adding EZ-Link Sulfo-NHS-LC-Biotin and antibody, the PBS buffer was incubated at room temperature for 60 min. The mixture was then dialysed with PBS buffer twice.

#### 2.2.4. Characterisation of Biotin-Antibody Conjugates

To estimate biotin incorporation, biotin-antibody solution was added to a mixture of HABA and avidin solution (Sigma, St. Louis, MO, USA). The biotin/antibody molar ratio was approximately 6.2, as calculated by the method described for the kit specification.

#### 2.2.5. Optimisation of Reaction Conditions

Osteocalcin in the blood is unstable. After secretion into the blood, intact osteocalcin is digested by proteases; therefore, there are two main forms of osteocalcin in serum: intact osteocalcin (amino acids 1–49) and the *N*-terminal fragment (amino acids 1–43). In this study, two special antibodies were employed to detect both forms. The essential reagents required for this assay include biotin-antibody, antibody-HRP conjugates, and streptavidin-coated magnetic particles. Upon mixing the antibody-HRP conjugates, a biotin-antibody, and a serum containing osteocalcin, a sandwich complex was formed. Then, streptavidin-coated magnetic particles were added to isolate the sandwich complex. The generated RLUs are proportional to the osteocalcin concentration. By utilising several different serum references of known osteocalcin concentrations, a concentration-response curve can be generated, and the osteocalcin concentration of an unknown can be ascertained. In this study, factors such as sample size, biotin-antibody and antibody-HRP concentrations, and incubation time were optimised.

## 3. Performance Test Method

### 3.1. Limit of Detection (LoD)

The calibrator was tested 20 times with 0 ng/mL analyte, after which the mean + 3SD was calculated. The corresponding concentration was considered the LoD.

### 3.2. Precision and Recovery

Three serum samples of different osteocalcin concentrations were tested and duplicated separately in one experiment and repeated after 20 days, and the intra-assay and interassay coefficient of variation (CV) were calculated. Concentrated osteocalcin solutions were added to three serum samples with different analyte levels, and the recovery rate was calculated.

### 3.3. Cross-Reactivity (CR)

The specificity of the osteocalcin antibody used to select substances was evaluated by adding the interfering substance to a serum matrix at various concentrations. CR is defined as the point where the reduction in signal corresponds to 50% of the signal achieved in the absence of analyte (B/B0 of 50%) and presented as a percentage of the analyte concentration given the same decrease in signal. The CR values were calculated as follows:

(1) $$\begin{array}{c} \text{CR}\left(\text{\%}\right)=\frac{\text{IC}50\text{ of osteocalcin}}{\text{IC}50\text{ of competitor }}\times 100\text{\%.} \end{array}$$

### 3.4. Accelerated Stability

The whole kit, including biotinylated antibody, SA-coated magnetic particles, antibody-HRP conjugate, and osteocalcin standards, was incubated at 37°C for 7 days to compare standard and sample signals on different days.

### 3.5. Method Comparison

The established method was compared with the market CLIA method from Roche. In total, 171 samples were used, ranging from 0.6 to 283.3 ng/mL.

### 3.6. Hook Effect

Concentrated osteocalcin solutions and the highest calibrator level were tested, and then the mean − 3SD value of the concentrated osteocalcin and mean + 3SD of the highest calibrator level were calculated and compared.

## 4. Results and Discussion

### 4.1. Advice on Development of the CLIA Method

#### 4.1.1. Method Procedure

First, 10 *μ*L serum, 120 *μ*L biotinylated antibody, and 120 *μ*L antibody-HRP conjugate were added to the reaction tube, mixed, and incubated for 16 min at 37°C. Next, 30 *μ*L streptavidin-coated magnetic solution was added and incubated for another 6 min at 37°C. After incubation, the magnetic particles were washed three times with washing buffer, and then 200 *μ*L substrate reagent was added to generate signals. A signal reader should be used to collect the RLUs and calculate the concentration values if a calibrator curve is available.

*(1) Optimisation of Sample Size, Biotin-Antibody, and Antibody-HRP Concentration*. *Hook Effect Observation*. In the initial phase of this study, serum samples with high osteocalcin concentrations usually showed poor linearity. After gradient dilution of these samples, a nonlinear relationship became evident, indicating the presence of the hook effect, as summarised in Table 1.

**Figure 2:**
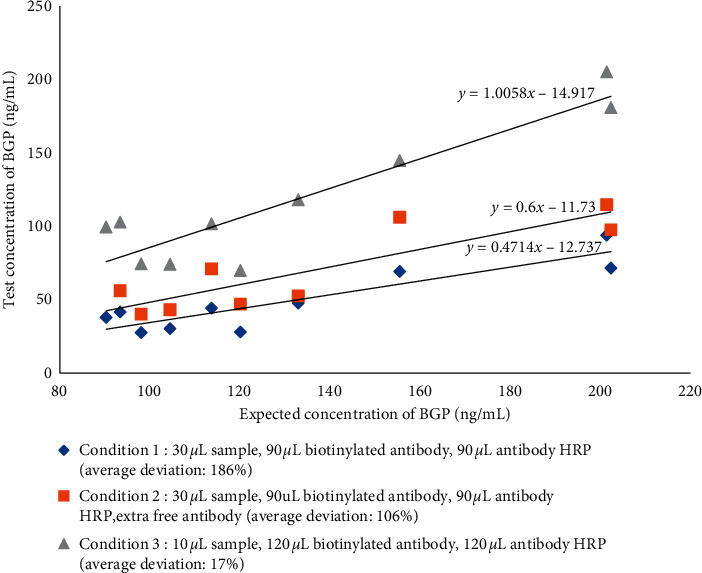
Optimisation of the reaction conditions.

*(2) Optimisation of the Sample Volume and Antibody Concentration in the Reagent*. To resolve the previously observed hook effect, several strategies were used to optimise the conditions. The antibody concentration was increased in the reaction system to match that of the analyte. As shown in Table 2, a series of concentrations of biotinylated antibody and antibody-HRP were tested, the signal (250 ng/mL)/ signal (0 ng/mL) values were greatest when the biotinylated antibody and antibody-HRP concentrations were 0.5 and 2 *μ*g/mL, respectively. Additional free antibodies were added to the reaction system to consume excess antigen in samples. The sample volume and reaction volume were also optimised. As shown in Figure 2, the best correlation between the expected and tested concentrations was observed with the third condition, which simultaneously reduced the sample volume and increased the reaction volume. Finally, 10 *μ*L serum, 120 *μ*L biotinylated antibody (0.5 *μ*g/mL), and 120 *μ*L antibody-HRP conjugate (2 *μ*g/mL) were selected as the optimal formation.

**Figure 3:**
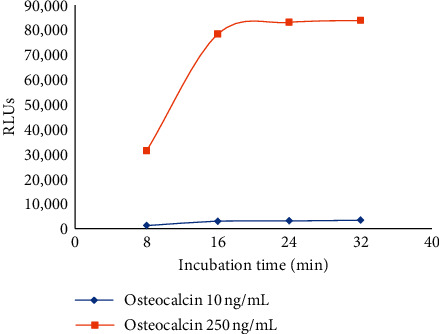
Optimisation of incubation time.

*(3) Optimisation of Incubation Time*. Biotinylated antibody and antibody-HRP incubation times were assessed. Aside from the incubation time, the procedure remained as described in the methods. RLUs increased with the extension of incubation time and remained unchanged after 16 min when the reaction reached dynamic equilibrium. Due to time-saving clinical requirements, we selected a 16 min incubation time. The signal results are shown in Figure 3.

#### 4.1.2. Method Performance

**Figure 4:**
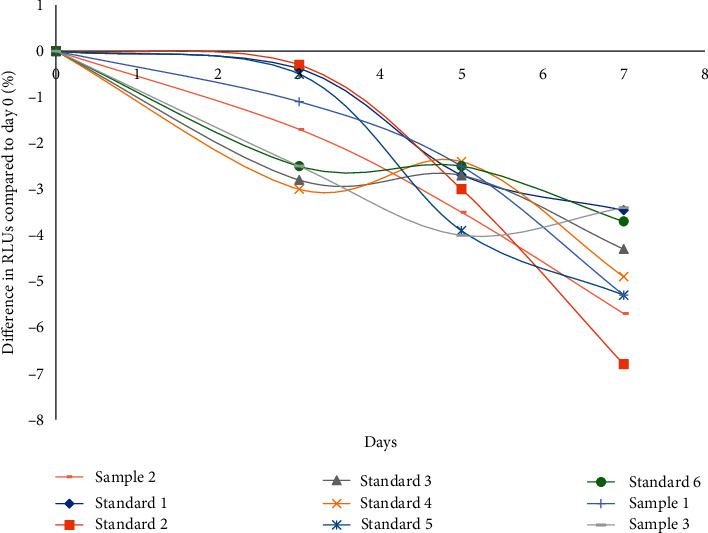
Accelerated stability results of reagent kits at 37°C.

The bone Gla protein (BGP) standard curve was fit by a 4-parameter logic function method. The LoD was 0.029 ng/ml. The precision testing results revealed intra- and interassay CVs of 3.22%–5.64% and 4.42%–7.25%, respectively (Table 3). The recovery rate was 96.53%–107.15% (Table 4). The developed methods exhibited high selectivity for BGP. Cross-reactivity was less than 0.1% with bovine, rat, mouse, rabbit, or porcine samples. An accelerated stability study revealed that the developed reagent showed great stability under temperature stress. The RLUs changed by less than 10% during the 7-day period at less than 37°C (Figure 4).

#### 4.1.3. Comparison of Methods

**Figure 5:**
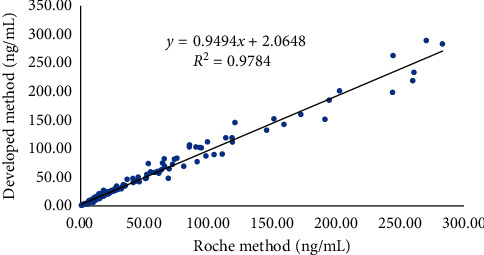
Comparison of the developed method and Roche method.

In this study, 171 serum samples were measured using both the developed method and the Roche method. The test results were regressed using the least-squares regression equation, and the correlation coefficient was computed. Data are shown in Figure 5. The test results show good agreement between the developed and compared methods, and the difference in test and mean values indicated a slight bias between these methods.

#### 4.1.4. Hook Effect

Patient serum was spiked with concentrated osteocalcin to prepare a series of hook samples. Hook samples and the highest calibrator level were tested (Table 5). The RLUs of concentrated osteocalcin were larger than those of the highest calibrator, indicating that there was no hook effect even at BGP concentrations as high as 4000 ng/mL.

#### 4.1.5. Clinical Study

**Figure 6:**
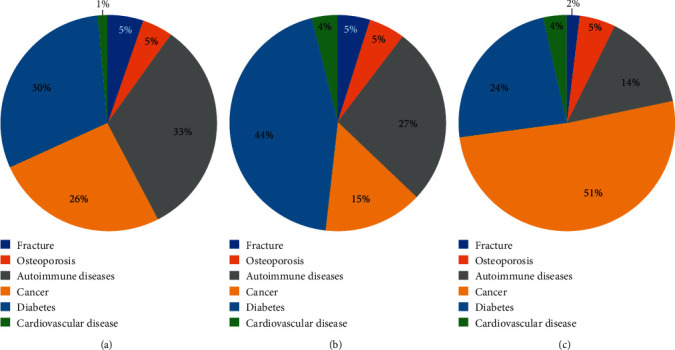
Distribution of clinical samples from postmenopausal women. (a) BGP <10 ng/mL. (b) BGP within 10–30 ng/mL. (c) BGP >30 ng/mL.

**Figure 7:**
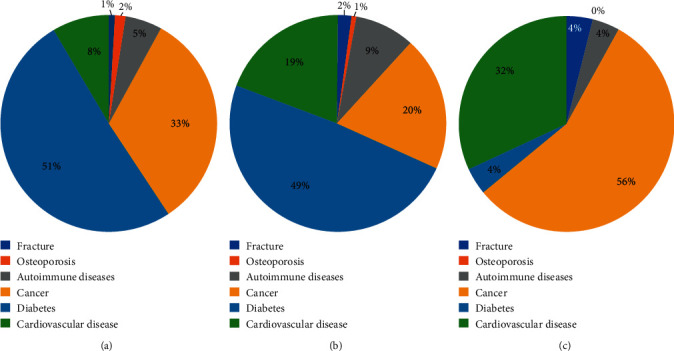
Distribution of clinical samples from men. (a) BGP <10 ng/mL. (b) BGP within 10–40 ng/mL. (c) BGP >40 ng/mL.

**Figure 8:**
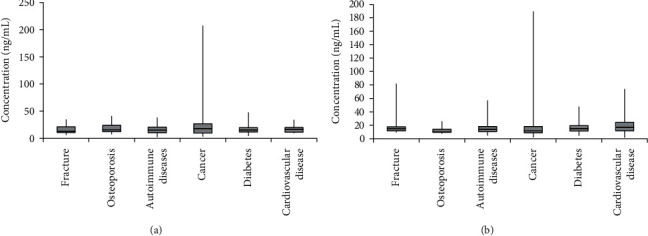
BGP concentration in postmenopausal women (a) and BGP concentration in men (b) .

In total, 1069 clinical patient samples were measured using the reagent kit developed in this study. Six conditions were involved (Table 6). The distribution of BGP differed between men and postmenopausal women (Figures 6 and 7). The range of BGP concentrations in different patients is shown in Figure 8.

## 5. Conclusions

The hook effect is commonly observed in experiments due to excessively high concentrations of analytes with both capture and detector antibodies. The hook effect occurs mostly (but not exclusively) in one-step immunometric (sandwich) assays, resulting in a decrease in the signal at very high analyte concentrations [14]. For some immunoassays, if there are large analyte concentration ranges (such as with ferritin, growth hormone, hCG, PRL, Tg, and the tumour markers PSA, CA199, and CA125), antigen-antibody reactions can lead to an antigen excess, resulting in falsely decreased results and potential misdiagnosis [14]. But in a direct competitive immunoassay, the hook effect is not exited [15]. According to the previous literature, there are numerous BGP quantification methods, such as the competitive and two-site radioimmunoassay [11], enzyme-linked immunosorbent assay with monoclonal antibodies [16] or polyclonal antibodies [17], and dual-label immunofluorometric assay [18]. However, these methods are time-consuming and have low throughput, thus limiting wide application, especially in institutions with large sample sizes. This study establishes a CLIA for the quantification of BGP in human serum with good performance and overall stability. Comparison of the methods demonstrated that this method has a good correlation with the reagent kit from Roche, which is widely used on the market. During the development of this method, a phenomenon was noticed: the serum of some patients showed a very high BGP concentration but low results. This hook effect provides false information to doctors. We decreased the sample volume and adjusted the antibody concentration to prevent such a situation. Consequently, no further false results were found. Here, biomaterials produced in-house, such as biotinylated antibody and antibody-HRP conjugate, were critical to the reagent performance. Therefore, further studies should be conducted on procedures for producing antibodies and conjugation. Minimising the differences in raw materials between batches should help to sustain the performance of the reagent kit. The clinical study using the developed kits showed good agreement between test results and disease, and the reagent kit fulfilled the clinical requirements.

## Figures and Tables

**Table 1:** Osteocalcin concentrations in serum samples after several dilutions.

Dilution	Sample 1	Sample 2	Sample 3
	ng/mL		
None	63.5	96.5	67.8
1 : 1	85.6	154.7	113.4
1 : 3	74.1	141.1	102.2
1 : 5	51.4	90.1	69.1
1 : 7	29.8	56.5	42.3
1 : 15	16.2	32.0	23.6

**Table 2:** Optimisation of antibody concentration.

Biotinylated antibody (*μ*g/mL)	0.5			1			1.5		
Antibody-HRP (*μ*g/mL)	2	1	0.5	2	1	0.5	2	1	0.5
Standard 1 (0 ng/mL)	288	213	164	268	215	153	260	217	164
Standard 2 (10 ng/mL)	3009	1986	1685	2769	2145	1634	3046	2059	1748
Standard 3 (25 ng/mL)	9855	6110	5420	10151	6538	5420	10963	7126	4932
Standard 4 (50 ng/mL)	26158	17526	13341	24327	18928	13074	22867	18928	13858
Standard 5 (75 ng/mL)	35566	18850	15649	39123	18285	14710	36776	17188	14416
Standard 6 (250 ng/mL)	76489	39010	36715	80314	40961	39652	83527	44238	36083

**Table 3:** Precision test results.

Samples	Intra-assay CV (*n* = 20)			Interassay CV (*n* = 20)		
	Mean (ng/mL)	SD	CV (%)	Mean (ng/mL)	SD	CV (%)
1	19.98	1.09	5.46	19.32	1.4	7.25
2	45.53	2.03	4.46	44.83	2.64	5.89
3	80.87	2.6	3.22	82.13	3.63	4.42

**Table 4:** Analytical recovery.

	BGP concentration (ng/mL)			
Samples	Added concentration	Tested	Expected	Recovery (%)
1	0.00	19.98	21.03	105.26
	15.63	35.57	34.93	98.20
	31.25	49.39	49.95	101.13
	62.50	81.42	83.48	102.53
	125.00	136.48	144.23	105.68

2	0	44.76	45.80	102.32
	15.63	59.18	61.04	103.14
	31.25	78.32	75.60	96.53
	62.50	105.01	105.39	100.36
	125.00	157.39	168.64	107.15

**Table 5:** RLUs of concentrated osteocalcin samples.

Conc. of BGP (ng/mL)	Mean	SD	Mean ± 3 *∗* SD
250	805,635	40,193	926,213
1000	1,377,486	88,866	1,644,083
2000	1,654,702	59,349	1,832,748
4000	1,807,267	47,002	1,948,274

**Table 6:** Distribution of clinical samples.

Populations	Conc. of BGP (ng/mL)	Fracture	Osteoporosis	Autoimmune diseases	Cancer	Diabetes	Cardiovascular disease
Postmenopausal female	<10	7	6	43	34	40	2
	10∼30	21	24	117	65	194	17
	>30	1	3	8	28	13	2

Male	<10	1	2	7	43	66	11
	10∼40	6	2	26	58	141	56
	>40	1	0	1	14	1	8

## Data Availability

The data used to support the findings of this study are available from the corresponding author upon request.
